# Sparse Analyzer Tool for Biomedical Signals

**DOI:** 10.3390/s20092602

**Published:** 2020-05-02

**Authors:** Stefan Vujović, Andjela Draganić, Maja Lakičević Žarić, Irena Orović, Miloš Daković, Marko Beko, Srdjan Stanković

**Affiliations:** 1 Faculty of Electrical Engineering, University of Montenegro, 81000 Podgorica, Montenegro; stefanv@ucg.ac.me (S.V.); majal@ucg.ac.me (M.L.Ž.); irenao@ucg.ac.me (I.O.); milos@ucg.ac.me (M.D.); srdjan@ucg.ac.me (S.S.); 2 COPELABS, Universidade Lusófona de Humanidades e Tecnologias, 1700-097 Lisboa, Portugal; beko.marko@ulusofona.pt; 3 UNINOVA, Faculdade de Ciências e Tecnologia, 2829-517 Monte Caparica, Portugal

**Keywords:** biomedical signals, compressive sensing, concentration measure, gradient algorithm, OMP, SIRA, statistical analyzer, sparse signal processing, TV minimization, virtual instrument

## Abstract

The virtual (software) instrument with a statistical analyzer for testing algorithms for biomedical signals’ recovery in compressive sensing (CS) scenario is presented. Various CS reconstruction algorithms are implemented with the aim to be applicable for different types of biomedical signals and different applications with under-sampled data. Incomplete sampling/sensing can be considered as a sort of signal damage, where missing data can occur as a result of noise or the incomplete signal acquisition procedure. Many approaches for recovering the missing signal parts have been developed, depending on the signal nature. Here, several approaches and their applications are presented for medical signals and images. The possibility to analyze results using different statistical parameters is provided, with the aim to choose the most suitable approach for a specific application. The instrument provides manifold possibilities such as fitting different parameters for the considered signal and testing the efficiency under different percentages of missing data. The reconstruction accuracy is measured by the mean square error (MSE) between original and reconstructed signal. Computational time is important from the aspect of power requirements, thus enabling the selection of a suitable algorithm. The instrument contains its own signal database, but there is also the possibility to load any external data for analysis.

## 1. Introduction

The processing of under-sampled signals has attracted significant research interest in the last decade [1,2,3,4,5,6,7,8,9,10]. The signal is under-sampled if the number of available samples is less than the number of samples required by the Shannon Nyquist sampling theorem. The under-sampling can be done intentionally during the acquisition procedure or signal samples could be lost during the transmission or discarded owing to noise. Intentional omitting of signal samples and their later reconstruction found its usage in the applications dealing with a large number of signal samples, with the purpose of increasing the processing speed. Such an approach could be important in medical applications. For example, in magnetic resonance imaging (MRI), lowering the required amount of data reduces the time of patient exposure to the harmful MR waves [11].

With the increasing use of wireless technology and smart devices in our everyday life, portable medical devices become very popular [12,13,14]. Modern technology is changing the way medicine approaches various health problems in a number of diseases and for a large number of patients. Using portable medical devices, the possibility to monitor patient condition every moment and in every place is provided. Technology can ease life to many patients, especially those with chronic diseases, patients with diabetes, those with cardiovascular diseases, or elderly people [13]. For example, automatic reminders for taking medications are developed, as well as devices for monitoring different health parameters [14,15] such as body temperature, glucose levels, blood pressure, and so on. Real-time monitoring and on-time reactions can save the patient’s life in situations when there is no need for hospitalization, but the vital parameters should constantly be tracked. Monitoring in such a way improves patient comfort, while at the same time unloading the hospital capacities in terms of staff and space [13].

Another important aspect of digital monitoring is the opportunity to store collected data in the patient’s medical record, from where it can be easily transferred to hospitals and clinicians all over the world. The diagnosis could be provided at a distance, without having physical contact with the patient. This has numerous advantages: avoiding going to the hospitals and waiting, getting opinions from many professionals around the world, and so on.

The communication between the patient and the healthcare professional should be fast and secure. The intentional under-sampling of medical data could lead to dealing with a much smaller amount of data, and thus faster transmission [11]. Moreover, sending only part of the original signal keeps the information sent secure. At the receiver part, the under-sampled signal should be recovered and back to its original version. The special problem during the transmission could be noise. Noisy samples could be considered as missing and, if they are detected, lead to signal under-sampling [4].

One of the new areas that enables under-sampled signal recovery is called compressive sensing (CS). The idea behind the concept of CS is to reduce the sampling rate far below that defined by the Nyquist–Shannon sampling theorem, and later recover the whole signal by applying complex mathematical algorithms [2,4,11,16,17,18,19,20,21,22,23,24,25,26,27,28,29,30,31,32]. Therefore, the CS represents a completely new paradigm compared with the traditional sensing strategy, and has been used in various applications such as image processing, biomedical signals, wireless communications, and radars [26,27,28,29,30]. In the CS scenario, the signal can be completely recovered from a small set of randomly acquired linear measurements, if the signal of interest has a sparse representation when represented in a certain transformation basis. Sparse representation means that the signal can be represented by a few non-zero coefficients, which is much lower than its original dimension. Consequently, for signals in real-world applications, it is important to identify sparse representations using the appropriate dictionaries of atoms or transformations.

Here, the application of this attractive approach to the various signals in biomedicine will be presented, providing clinicians and researchers with a good base for possible improvement of different technology-based services and devices used in medicine. The proposed instrument implements a number of algorithms in order to perform reconstruction, as well as to compare the results obtained with different algorithms. This tool can be used to create a number of different signals, with a specially designed panel where all important signal parameters can be easily chosen, such as signal sparsity, percent of missing samples, signal length, and so on. Finally, the paper combines several algorithms that solve the reconstruction problem using quite different mathematical approaches, also contributing to the educational dimension of the proposed instrument. The software provides the possibility to compare several different algorithms in terms of reconstruction precision (expressed in terms of mean square error (MSE)) and achieved sparsity level (expressed in terms of concentration measures *ℓ*_1_-norm and Gini index). On the basis of the comparison results, the user chooses the transform and the algorithm that best suit the considered signal type. Moreover, the user can choose the solution that provides the best sparsity, or the lowest reconstruction error, or as a third option, a solution providing the best trade-off between these two measures.

By providing the analytical visualization of results, together with reconstruction error and concentration measures, the tool allows users to test and choose the best possible optimization approach and the most suitable sparse domain for the applications with biomedical signals or images. Namely, among a number of algorithms for sparse signal recovery and transform domain modeling, the proposed tool allows the selection of an appropriate approach combined with the suitable transformation for achieving the most accurate reconstruction results for the considered signal types. The tool is designed in a user-friendly manner even for the non-specialists in the field, as it does not require certain pre-knowledge about the implemented approaches. On the basis of the statistical parameters for measuring reconstruction efficiency, the users are able to choose the most suitable among the offered solution. However, it is also convenient for the researchers working in the field, as it provides a set of comprehensive solutions for the processing of biomedical data that can be further extended or adapted for different purposes.

The paper is structured as follows. Section 2 provides a theoretical background on the CS. Section 3 describes approaches for signal recovery that are implemented within the instrument. Section 4 describes the software, while Section 5 gives the results applied to concrete biomedical signals and images. The conclusion is given in Section 6.

## 2. Theoretical Background

Consider a time-domain signal *x*(*n*), composed of *N* samples, that is, *n* = 0, 1, …, *N* − 1. Suppose that the arbitrary linear transform of this signal is denoted with *X*(*k*), where *k* = 0, 1, …, *N* − 1.

If the most of the coefficients of *X*(*k*) are zero-valued or negligible, then *X*(*k*) represents a sparse presentation of signal *x*(*n*). For a *K*-sparse signal, it might be said that only *K* coefficients of signal *X*(*k*) are non-zero, where *K* << *N*.

Signal *x*(*n*) and *X*(*k*) are related via the following [2,4]:

(1) $$X(k)=\sum_{n=0}^{N-1}x(n)\psi_{k}^{*}(n),\;x(n)=\frac{1}{N}\sum_{k=0}^{N-1}X(k)\psi_{k}(n),$$

where ***ψ****_k_*(*n*) and its inverse ***ψ***_k_*(*n*) are basis functions. In the case of the discrete fourier transform (DFT) as one of the commonly used transforms, the basis function equals ***ψ****_k_*(*n*) = *e^j^*^2π*nk*/*N*^. Note that, in general, CS algorithms deal with arbitrary linear transformations like discrete cosine transform (DCT), Hermitian transform (HT), wavelet transform [33,34], and so on, while some of the algorithms like the gradient-based one [11,32] deal even with nonlinear transformations. In this work, the HT, DCT, and DFT transforms are considered, as they found many applications in biomedical data representation and analysis [35,36,37,38,39]. For example, DFT is used for the analysis of electroencephalography (EEG) signals and blood pressure signals [35], and DCT and DFT transforms are used in MRI image processing [36,37], while the HT is used in electrocardiograph (ECG) and QRS signal analysis [38,39].

Discrete Hermite function of an order *p* is defined as follows [5,6]:

(2) $$\psi_{p}(t)=\frac{e^{-\frac{t^{2}}{2\lambda^{2}}}H_{p}\left(t/\lambda \right)}{\sqrt{\lambda 2^{p}p!\sqrt{\pi }}},\;H_{p}(t)={\left(-1\right)}^{p}e^{t^{2}}\frac{d^{p}(e^{-t^{2}})}{dt^{p}},$$

where *H_p_* is a Hermite polynomial of the *p*-th order, *λ* is the scaling factor used for “stretching” or “compressing” the Hermite functions, while the HT matrix can be defined as follows:

(3) $$\Psi =\frac{1}{M}\left[\begin{array}{ccc} \frac{\psi_{0}(n_{1})}{{\left(\psi_{M-1}(n_{1})\right)}^{2}} & \vdots & \frac{\psi_{0}(n_{M})}{{\left(\psi_{M-1}(n_{M})\right)}^{2}}\\ \frac{\psi_{1}(n_{1})}{{\left(\psi_{M-1}(n_{1})\right)}^{2}} & \vdots & \frac{\psi_{1}(n_{M})}{{\left(\psi_{M-1}(n_{M})\right)}^{2}}\\ \ldots & \vdots & \ldots \\ \frac{\psi_{M-1}(n_{1})}{{\left(\psi_{M-1}(n_{1})\right)}^{2}} & \vdots & \frac{\psi_{M-1}(n_{M})}{{\left(\psi_{M-1}(n_{M})\right)}^{2}} \end{array}\right].$$

The “stretching” or “compressing” of the Hermite functions is used for better fitting of the function to the signal [38], providing better sparsity of the signal in the HT domain.

When dealing with the HT, signal *x*(*n*) should be sampled at the non-uniform points that correspond to the roots of the Hermite polynomial. Another approach is to interpolate the uniformly sampled signal in order to obtain requested signal values at the non-uniform points. Hermite functions obtained by the uniform sampling of the corresponding continuous functions are not orthogonal. Therefore, in order to obtain the orthogonal function, the interpolation of the Hermite function is used. The second approach, interpolation, is also implemented within the instrument. The signal sampled according to the sampling theorem is interpolated using the sinc interpolation formula [11,27]:

(4) $$x(\sigma n_{m})\approx \sum_{i=-K}^{K}x(iT)\frac{\sin\left(\pi (\sigma n_{m}-iT)/T\right)}{\pi (\sigma n_{m}-iT)/T},\;m=1,\ldots ,M.$$

where *T* denotes the sampling period. The optimal value of the parameter *σ* produces the best possible concentration in the transform domain, and it can be found using the *ℓ*_1_-norm optimization:

(5) $$\sigma_{opt}\underset{\sigma }{=min}{\left\|HT\left\{x(\sigma n_{m})\right\}\right\|}_{1}.$$

The instrument implements several commonly used sparsity measures that are suitable for the observed signals. Besides the *ℓ*_1_-norm concentration, there are a lot of approaches for measuring sparsity, for example, entropy based approaches [40,41] or the Gini index [41,42,43,44]. The Gini index satisfies most of the desirable characteristics of measures of sparsity and overcomes the limitations of standard norm-based sparsity measures, as proven in [41]. It is suitable for comparing the sparsity of a signal in different transform domains [42], and is also used as a measure of sparsity for biomedical signals [43,44]; therefore, this measure is implemented within the instrument together with the *ℓ*_1_-norm concentration. The Gini index is calculated according to the following relation:

(6) $$G(x)=1-2\sum_{i=1}^{N}\frac{\left|x_{s}(i)\right|}{{\left\|x\right\|}_{1}}\left(\frac{N-i+\frac{1}{2}}{N}\right),$$

where *x_s_* is sorted version of a set of elements in ascending order. The Gini index values can be between 0 and 1. A higher value of the Gini index corresponds to better sparsity.

Here, the HT transform is mainly implemented for the application in electrocardiograph (ECG) signals and their QRS complexes. An ECG signal represents the electrical activity of a heart over time, while the QRS complex is formed by three of the graphic deflections of the ECG signal [45]. The analysis of the ECG signals and QRS complexes is used in heart function monitoring and disease diagnostic. The interval between two successive QRS provides information about the regularity of the cardiac rhythm. Moreover, the QRS observation is used in the diagnosis of other heart abnormalities such as myocardial infarction or arrhythmia and, therefore, the QRS represents an important part of the ECG signal. Having in mind that the Hermite functions show physical similarity with QRS complexes, they are found to be a suitable tool for their analysis [6,38,46].

Another observed transformation, DCT, has the following form:

(7) $$DCT(k)=c(k)\sum_{n=0}^{N-1}x(n)\cos\frac{(2n+1)k\pi }{2N},$$

where *k* = 0, …, *N* − 1 and *c*(*k*) is as follows:

(8) $$c(k)=\{\begin{array}{c} \sqrt{1/N},\;k=0\\ \sqrt{2/N},\;k=1,\ldots ,N-1 \end{array}.$$

In the matrix notation, the above equations can be generally written as follows: **X** = **Ψx** and **x** = **Ψ**^−1^**X**, where the vector **X** has elements *X*(*k*), and vector **x** has elements *x*(*n*). Both vectors are of length *N*, and **Ψ** is *N × N* transform matrix with elements ***ψ***_k_*(*n*).

Suppose an *M*-length vector **y**, which is a linear combination of elements from vector **X**. This vector is obtained as follows [4]:

(9) y=AX,

where **A** is an *M* × *N* matrix. The CS task is to reconstruct signal **X** (or **x**) from vector **y**. Note that the length of vector **y** is lower than the length of **X**, because (*M* < *N*). Construction of matrix **A** attracts significant research interest. Namely, the randomly constructed sensing matrix is considered in all implemented algorithms. The sensing matrix is a random matrix **Φ** that is multiplied by the transform matrix **Ψ**, resulting in the compressive sensing matrix **A**. The compressive sensing matrix **A** is called the random partial transform domain matrix containing the random combination of rows from **Ψ** that corresponds to the random position of the available samples. The vector **y** of length *M* is equal to samples of signal **x**, taken at the positions corresponding to preserved rows in matrix **Ψ**^−1^, that is,

(10) $$y(i)=x(n_{i}),\;i=1,2,\ldots ,M.$$

The reconstruction error may depend on the choice of the domain of sparsity and reconstruction algorithm, but in certain cases, it may also be the consequence of the quantization influence, as discussed in [47]. Namely, the limitations of the number of bits used for the representation of the available signal samples can affect the reconstruction performance. If the measurements *y*(*m*) are normalized to the range −1 ≤ *y*(*m*) ≤ 1, and the *B*-bit registers are assumed, then sparse coefficients *X*(*i*) have to be within the range $-\min\{\sqrt{M/K},1\}<X(i)<\min\{\sqrt{M/K},1\}$, in order for product **y** = **AX** to produce amplitudes below 1 [47]. The reconstruction error related to the number of bits is given by [47] *e*^2^ = 3.01 × log_2_*K* − 6.02*B* − 7.78. The proposed solution is designed for the 64-bit computer device, so the effects of quantization in this case can be considered negligible. However, the quantization issues should be carefully considered especially for hardware realizations of considered reconstruction algorithms.

## 3. Approaches for Under-Sampled Signal Reconstruction

Another direction within the CS area is related to the reconstruction algorithms for compressed sensed data. Many signal reconstruction algorithms have been proposed depending on the type of signal and CS scenario [4,11,16,17,18,19,20,21,22,23,24,25,26,27,28,29,30,31,32]. The performance of these algorithms may vary depending on the number of missing samples, level of sparsity, and amount of external noise, and there is still a lack of general instructions for their practical use.

Among the algorithms for 1D signals’ reconstruction that are included in the Virtual instrument, the *ℓ*_1_-magic algorithm is included from the class of convex algorithms. Next, the orthogonal matching pursuit (OMP) algorithm [21] from the class of greedy algorithms is implemented, as well as the single iteration construction algorithm (SIRA), based on the analytical threshold derivations [9,11,26] and generalized deviation-based reconstruction algorithm [23]. Finally, as an efficient and simple solution, the gradient-based convex algorithm is implemented [32]. This algorithm suits for a general class of signals, and for both 1D and 2D cases. It can be successfully used when the measurements are affected by the noise and also provides satisfactory results for the natural images reconstruction from a very reduced set of pixels. Greedy approaches such as SIRA, OMP, and generalized deviation-based reconstruction algorithm (GDBRA) are faster, but less precise compared with convex optimization algorithms and also require a priori knowledge about the signal (e.g., a number of components). The implemented reconstruction algorithms are briefly described in the sequel.

### 3.1. L1-Magic

The solution of problem (9) requires exhaustive searches over subsets of columns of the CS matrix A and, therefore, is not computationally feasible. Computationally more suitable approaches solve a convex optimization problem through linear programming, such as basis pursuit (BP), basis pursuit de-noising (BPDN), least angle regression (LARS), least absolute shrinkage and selection operator (LASSO) [4,11,18,21,22], and so on. The near-optimal approach is provided using the convex ℓ_1_-minimization. It is defined as follows:

(11) $$\min{\left\|X\right\|}_{1}\;\mathrm{subject}\;\mathrm{to}\;y=AX.$$

Standard linear program form can be recast as follows [22]:

(12) $$\underset{X}{\min}\langle c_{0},X\rangle \;\mathrm{subject}\;\mathrm{to}\;y=AX,\;f_{i}(X)\leq 0.$$

Each of $f_{i}(X)=\langle c_{i},X\rangle +d_{i},\;i=1,\ldots ,m$ is a linear functional for $c_{i}\in R^{N},\;d_{i}\in R$. At the optimal point, there will exist dual vectors $\nu^{*}\in R^{K}$ and $\lambda^{*}\in R^{M}$ that satisfy Karush–Kuhn–Tucker conditions:

(13) $$\begin{array}{ll} c_{0}+A^{T}\nu^{*}+\sum_{i}\lambda_{i}{}^{*}c_{i}=0, & \lambda_{i}^{*}f_{i}(\tilde{X})=0,\;i=1,\ldots ,m\\ & A\tilde{X}=y,\;f_{i}(\tilde{X})\leq 0,\;i=1,\ldots ,m. \end{array}$$

The algorithm finds the needed vector (optimal dual vector) by solving the system of nonlinear equations. At the interior point $\left(\tilde{X},\;\nu^{*},\;\lambda^{*}\right)$, the system is linearized and solved.

### 3.2. Gradient-Based Algorithm

The recently proposed gradient algorithm [32] for sparse signal reconstruction is a computationally efficient algorithm that belongs to a wide class of gradient CS algorithms. The idea behind this algorithm is to observe missing samples in a dense domain as the variables, which are reconstructed in a way to produce minimal concentration measure in the sparse domain. Reconstruction of missing samples is the main difference of this algorithm compared with the others, which mainly reconstruct the signal in the sparse domain. The implementation steps for the 1D signal are given in Algorithm 1.

### 3.3. SIRA—Single Iteration Reconstruction Algorithm

Single iteration reconstruction algorithm [3,6,7,25] is based on the idea of separating signal components in the sparsity domain from the noise components that appear as the consequence of missing samples. The required assumption is that all signal components are above the calculated threshold, while the noise components are under the threshold. The probability that values corresponding to noise are lower than *T* is *P*(*T*).
**Algorithm 1.** Gradient-based algorithm**Input**: set of the positions of the missing samples: ${\mathbb{N}}_{x}$; measurement vector **y**;    Set $x^{\left(0\right)}(n)\leftarrow y(n)$ for $n\notin {\mathbb{N}}_{x}$    Set $x^{\left(0\right)}(n)\leftarrow 0$ for $n\in {\mathbb{N}}_{x}$    m←0    Set $\Delta \leftarrow \max\left|x^{\left(0\right)}(n)\right|$    **repeat**        **repeat**            $x^{\left(m+1\right)}(n)\leftarrow x^{\left(m\right)}(n)$, for each *n*            **for**
$n_{i}\in {\mathbb{N}}_{x}$
**do**       $\begin{array}{l} X^{+}(k)\leftarrow ℑ\{x^{\left(m\right)}(n)+\Delta \delta (n-n_{i})\},\\ X^{-}(k)\leftarrow ℑ\{x^{\left(m\right)}(n)-\Delta \delta (n-n_{i})\},\;\left(ℑ\;-\mathrm{transformation}\right) \end{array}$                    $g(n_{i})\leftarrow \frac{1}{N}\sum_{k=0}^{N-1}({\left\|X^{+}(k)\right\|}_{1}-{\left\|X^{-}(k)\right\|}_{1}),$    $x^{\left(m+1\right)}(n_{i})\leftarrow x^{\left(m\right)}(n_{i})-g(n_{i})$    **end for**    m←m+1    **until stopping criterion is satisfied**    Δ←Δ/3    **until required precision is achieved** **Output:** reconstructed signal $x^{\left(m\right)}(n)$

Depending on the sparsity domain, the threshold is calculated according to the relations derived in [6,7,25]. In the case of the DFT as a sparsity domain, the threshold *T_DFT_* is as follows:

(14) $$T_{DFT}=\sqrt{-\sigma_{MS}^{2}\log(1-P{\left(T\right)}^{\frac{1}{N-K}})}\approx \sqrt{-\sigma_{MS}^{2}\log(1-P{\left(T\right)}^{\frac{1}{N}})},$$

If the DCT is considered as a transformation domain, the threshold *T_DCT_* is calculated as follows:

(15) $$T_{DCT}=4\sqrt{\frac{M(N-M)}{N^{2}(N-1)}K},$$

while in the HT domain, the threshold *T_HT_* is follows:

(16) $$T_{HT}=\sigma_{MS}\sqrt{\left(-4/\pi -aL+\sqrt{{\left(4/\pi +aL\right)}^{2}-4aL}\right)/a},\;$$

where $a\leftarrow 0.147,\;L\leftarrow \log\left(1-{\left(P(T)\right)}^{2/M}\right)$ and *P*(*T*) is desired probability (e.g., 0.99).

In Equations (14)–(16), *M* denotes the number of missing samples, *N* is the signal length, and *K* is the sparsity. The parameter *N* − *K* in *T_DFT_* could be approximated as *N*, based on the fact that *K* << *N*. The steps of the algorithm are given in Algorithm 2. The resulting vector **X** contains the signal components values **X**_R_ at positions **k**, while the rest of the transform domain coefficients are zero.
**Algorithm 2.** SIRA**Input:**    Measurement vector **y**; *M* × *N* matrix **A**; signal sparsity *K*○Set desired probability (e.g., P←0.99)○Calculate variance—variance is calculated by using one of the relations, depending on the chosen transformation domain. The corresponding equations are in the following table:
***Transformation domain******DFT******DCT******HT******Variance***$\begin{array}{c} \;\sigma_{MS}^{2}=\\ =M\frac{N-M}{N-1}\sum_{i=1}^{M}\frac{y{\left(i\right)}^{2}}{M} \end{array}$$\begin{array}{c} \;\sigma_{MS}^{2}=\\ =M\frac{N-M}{N^{2}(N-1)}\sum_{i=1}^{K}A_{i}^{2}, \end{array}$$\begin{array}{c} \;\sigma_{MS}^{2}=\\ =\frac{(N-M)M-{\left(N-M\right)}^{2}}{M^{2}(M-1)}\sum_{i=1}^{K}A_{i}^{2} \end{array}$$\begin{array}{c} \;\sum_{i=1}^{K}A_{i}^{2}=\frac{N}{M}\sum_{n\in M}^{}s^{2}(n),\\ \;s(n)-\mathrm{available}\;\mathrm{samples} \end{array}$○For a given *P* calculate threshold according to relation (13);○$\mathrm{Calculate}\;\mathrm{the}\;\mathrm{initial}\;\mathrm{transform}\;\mathrm{domain}\;\mathrm{vector}\;X_{0}:\;X_{0}=A^{-1}y$;○$\mathrm{Find}\;\mathrm{positions}\;\mathrm{of}\;\mathrm{components}\;\mathrm{in}\;X\;\mathrm{higher}\;\mathrm{than}\;\mathrm{normalized}\;\mathrm{threshold}\;T,\;k=\arg\{\left|X_{0}\right|>T/N\}$;○$\mathrm{Form}\;\mathrm{CS}\;\mathrm{matrix}\;\mathrm{by}\;\mathrm{using}\;\mathrm{only}\;k\;\mathrm{columns}\;\mathrm{from}\;A,\;A_{CS}\leftarrow A(k)$○$\mathrm{Calculate}\;X_{R}={\left(A_{CS}^{T}A_{CS}\right)}^{-1}A_{CS}^{T}y$; **Output: X**_R_

### 3.4. GDBRA—Generalized Deviation-Based Reconstruction Algorithm

This algorithm uses the model of general deviations in the transform domain instead of transform representation itself. The algorithm works with signals sparse in the DFT domain, and the generalized deviations are derived for the DFT case. The use of generalized deviations is inspired by the robust statistics theory, where the form of transformation is adapted to the specific noise environment. Consequently, this approach provides flexibility of using different types of minimization norms for different noisy environments. The algorithm [23] is described through the steps in Algorithm 3.
**Algorithm 3.** GDBRA○**Step 1:** For each *k* = 0, 1, …, *N* − 1 calculate generalized deviation GD(*k*) as:
$$\begin{array}{l} GD(k)=\\ =\underset{n_{m}\in N_{avail}}{mean}\left\{{\left|x(n_{m})e^{-j2\pi kn_{m}/N}-\underset{n_{m}\in N_{avail}}{mean}\left\{x(n_{1})e^{-j2\pi \frac{kn_{1}}{N}},\ldots ,x(n_{M})e^{-j2\pi \frac{kn_{M}}{N}}\right\}\right|}^{L}\right\}, \end{array}$$
where $\underset{n_{m}\in N_{avail}}{mean}\{f(n_{M})\}$ is used to find mean value of vector with elements $f(n_{M})$, for $n_{M}\in Navail$. For norm *ℓ*_2_ use *L* = 2, while for norm *ℓ*_1_ use *L* = 1.○**Step 2:** Determine the signal support k=arg{GD(k)<T} where *T* can be calculated with respect to median{GD(k)}, or p⋅median{GD(k)} (where *p* is constant close to 1) for example, but also with respect to mean or minimal value. The vector of positions **k** should contain all signal frequencies $k_{i}\in k$ for any *I* = 1, …, *K*.○**Step 3:** Set $\tilde{X}(k)=0$ for frequencies k∉k;;○**Step 4:** The estimates of the DFT values can be calculated by solving the set of equations, at the localized frequencies from the vector **k**, where **k** contains *K* signal frequencies **k** = *k*_1_, *k*_2_, … *k_k_*. A system of equations is set as follows:$$\sum_{i=1}^{K}X(k_{i})e^{j2\pi k_{i}n_{m}}=x(n_{m})$$○**Step 5:** The CS matrix **A**_CS_ is formed as a partial DFT matrix: columns correspond to the positions of available samples, rows correspond to the selected frequencies. The system is solved in the least square sense:
$$X={\left(A_{CS}^{T}A_{CS}\right)}^{-1}A_{CS}^{T}y.$$

### 3.5. OMP—Orthogonal Matching Pursuit

Orthogonal matching pursuit (OMP) is a kind of greedy algorithm that finds the best correlation between measurements vector **y** and the matrix **A** through iterations. In each iteration, the column of **A** corresponding to certain sparse domain coefficient is found. The OMP implementation is given in Algorithm 4.

### 3.6. TV Minimization

Another approach, the total variation (TV) minimization, is implemented in the Virtual instrument. This approach can be successfully applied to both 1D and 2D signals. Generally, the images are not strictly sparse either in the spatial or in the space domain. Therefore, the *ℓ*_1_-minimization of the image gradient, named TV minimization, is found to be a more suitable approach than minimizing the image coefficients. This approach is suitable for noisy signals as well and is described with the following relation:

(17) $${\left\|x\right\|}_{TV}=\sum_{i,j}\left|{\left(\nabla x\right)}_{ij}\right|,\;\mathrm{where}\;\nabla_{i,j}x=\left[\begin{array}{l} x(i+1,j)-x(i,j)\\ x(i,j+1)-x(i,j) \end{array}\right]$$

In this paper, TV minimization is combined with the DFT and DCT transformations.
**Algorithm 4:** Orthogonal matching pursuit**Input:** Measurement vector **y**, *M* × *N* matrix **A**, signal sparsity *K*▪Set initial residual: $r_{0}\leftarrow y$▪Set initial indices: $\Omega_{0}\leftarrow \emptyset$▪Set matrix of chosen columns: $\Theta_{0}\leftarrow []$▪**for**i←1 to K    $\omega_{i}=\underset{j=1,\ldots ,N}{\arg\max}\left|\langle r_{i-1},A_{j}\rangle \right|$    - maximum correlation column    $\Omega_{i}\leftarrow \Omega_{i-1}\cup \omega_{i}$        - update set of indices    $\Theta_{i}\leftarrow \left[\Theta_{i-1}A_{\omega_{i}}\right]$        - update set of chosen columns    $x_{i}=\underset{x}{\arg\min}{\left|\left|r_{i-1}-\Theta_{i}x\right|\right|}_{2}^{2}$    $a_{i}\leftarrow \Theta_{i}x_{i}$    $r_{i}\leftarrow y-a_{i}$▪**end for**
**Output:**
**x_K_** and $\Omega_{K}$

### 3.7. Douglas–Rachford Algorithm

An efficient solution for 2D signal recovery is based on the Douglas–Rachford (DR) algorithm and its special case, alternating directions methods of multipliers (ADMM) [31,48]. The ADMM, that is, a variation on the method of multipliers, is a special case of Douglas–Rachford splitting. The DR problem is defined as follows [31,32,33,34,35,36,37,38,39,40,41,42,43,44,45,46,47,48]:

(18) $$\underset{x\in {\mathbb{R}}^{N}}{\mathrm{minimize}}\left(f(x)+g(x)\right),$$

where *f* and *g* are convex functions that should not be smooth, but only proximable. The proximal mappings for *f* and *g* are computed as follows [48]:

(19) $$prox_{\lambda f}(x)=\underset{y}{\arg\min}\frac{1}{2}{\left\|x-y\right\|}^{2}+\lambda f(y),\;prox_{\lambda g}(x)=\underset{y}{\arg\min}\frac{1}{2}{\left\|x-y\right\|}^{2}+\lambda g(y).$$

In CS terms, *f*(*x*) = *ŋ_H_* and $g(x)={\left\|x\right\|}_{1}$, where the affine space *H* is defined as *H* = {x: Ax = y}, while *ŋ_H_* is an indicator function [48]:

(20) $$\eta_{H}(x)=\left\{ \begin{array}{ll} 0 & \mathrm{if}\;x\in H,\\ +\infty & \mathrm{if}\;x\notin H. \end{array}\right..$$

Therefore, the proximal operators for functions *f*(*x*) and *g*(*x*) are as follows:

(21) $$\begin{array}{l} prox_{g}(x)=\max\left(0,1-\frac{\lambda }{\left|x_{}\right|}\right)x_{},\\ prox_{f}(x)=prox_{{\lambda \eta }_{H}}(x)=x+A^{T}{\left(AA^{T}\right)}^{-1}(y-Ax). \end{array}$$

The DR iteratively finds a solution according to the following relation:

(22) $${\tilde{x}}_{k+1}=(1-\mu /2){\tilde{x}}_{k}+\frac{\mu }{2}rprox_{\lambda g}(rprox_{\lambda f}({\tilde{x}}_{k})),\;0<\mu <2,$$

where *rprox* denotes reversed-proximal mappings given (for function *h*):

(23) $$rprox_{\lambda h}=2prox_{\lambda h}-h(x).$$

## 4. Design of the Software—Virtual Instrument for Biomedical Signals Reconstruction

**Figure 1:**
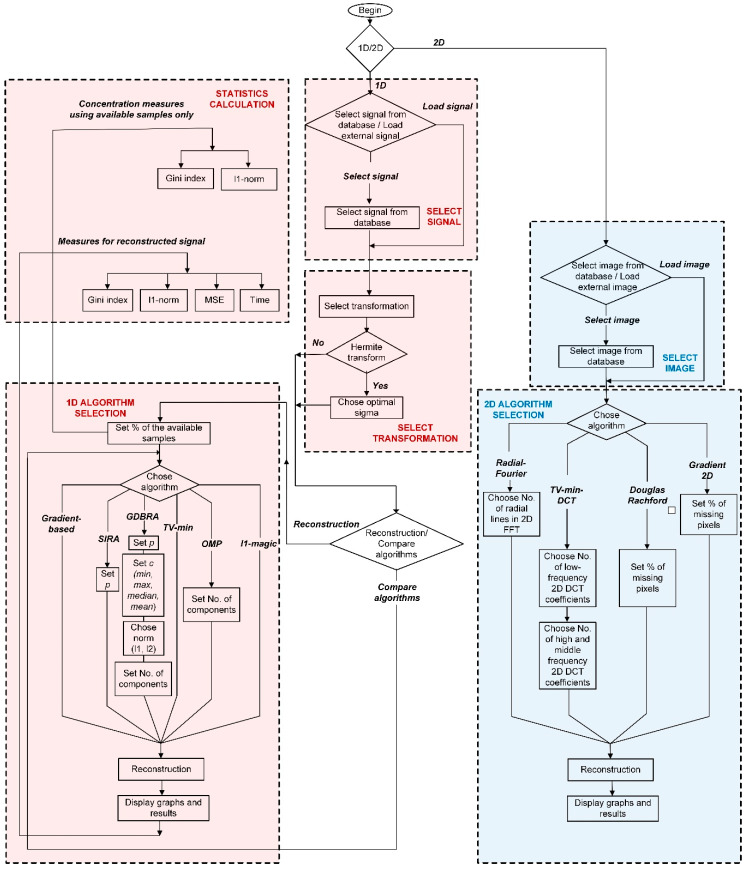
The flowchart for the virtual instrument realization. MSE, mean square error; SIRA, single iteration construction algorithm; OMP, orthogonal matching pursuit; GDBRA, generalized deviation-based reconstruction algorithm; DCT, discrete cosine transform; FFT, fast Fourier transform.

The detailed description of the developed software is described in the sequel. The proposed instrument is designed to work with various types of biomedical signals (both the 1D and 2D signals). The instrument is implemented in MATLAB 2017a version. The flowchart of the instrument is shown in Figure 1.

There is a specially designed panel where the required parameters for signal reconstruction can be easily chosen, for instance, the percent of missing samples, sparse transformation domain, and so on. The following reconstruction algorithms are implemented: *ℓ*_1_-magic algorithm and gradient-based algorithm from the convex optimization group, as well as the OMP algorithm, SIRA, and GDBRA from the greedy approaches group. The gradient-based approach is also suitable for 2D signals together with TV minimization, which belongs to the convex optimization group. Another 2D recovery approach is Douglas–Rachford splitting, implemented in several commonly used solvers for sparse reconstruction, such as YALL1, SALSA, and SpaRSA.

A special section of the Virtual instrument is devoted to the statistical analyzer, which provides some interesting statistical parameters for the evaluation of reconstruction algorithms, such as mean square error (MSE) and two concentration measures used for choosing the most suitable transform or the most suitable reconstruction algorithm. The second part of the proposed Virtual instrument is dedicated to 2D signals, that is, biomedical images. A number of images are provided in order to test several algorithms for sparse recovery.

### 4.1. Part 1—Reconstruction of 1D Signals

**Figure 2:**
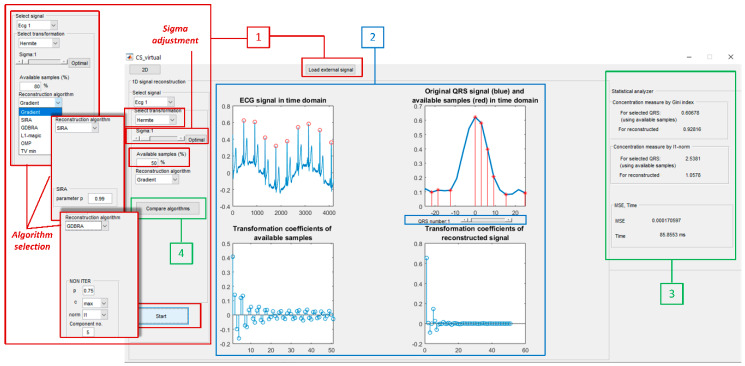
The outlook of part for 1D signal analysis. ECG, electrocardiograph.

The outlook of the solution for 1D signal recovery is presented in Figure 2. The instrument includes all described reconstruction algorithms and their relevant parameters, with the possibility for further extension with additional methods. In order to provide a user-friendly environment, the instrument is structured through few sections that will be explained in detail.

Section 1—Signal parameters adjustment: This part of the instrument is used to generate different types of signals in order to test implemented algorithms. The database contains ECG signals, extracted QRS complexes (there is also the option for users to extract QRS using the instrument), signals from respiration monitor, and brain activity signals such as electroencephalography (EEG) and electrooculography (EOG) signals. Some signals are taken from the open biomedical signals databases [49,50], while the others (e.g., respiration signal) are recorded using the National Instruments Elvis platform.

The first option within the instrument is to choose a signal from the drop-down menu, or to load a certain signal by using the *Load external signal* option. The measurements are obtained from time-domain samples, so there is an option to set a percentage of available data (e.g., time-domain samples). For example, if the percent of available samples is set to 25% for the signal length of 256 samples, then the length of measurement vector **y** will be 64, and the remaining 192 samples have to be recovered.

Furthermore, the options for sparse transform domain selection are provided. The transform can be selected from the drop-down menu. Selecting a transformation automatically calculates the concentration measure in the statistical analyzer panel. This is useful if the user does not have prior knowledge about the signal and its sparse transform domain. Two measures are provided, the *ℓ*_1_-norm and the Gini index, and these are calculated using the transform domain coefficients. The transformation that provides minimal *ℓ*_1_-norm or maximal value of Gini index is the sparsest among those observed. However, for choosing the most effective reconstruction algorithm, the MSE between the original and reconstructed signals should also be taken into account together with the concentration measures.

In the drop-down menu, the user can choose one of the commonly used transform basis: DFT, DCT, or HT. These are shown to be suitable for most of the considered biomedical signals [5,11,46]. It is important to note that, for the QRS complexes, the most suitable transformation is HT, owing to the similarity between the QRS signals and the Hermite functions. Therefore, for QRS, there is an option to optimize HT by choosing the sigma parameter.

In this part of the Virtual instrument, there is also a drop-down menu for choosing one of the offered algorithms. On the basis of the chosen algorithm, the panel changes and additional options appear, showing algorithm input parameters. Gradient and *ℓ*_1_-magic algorithms do not need any additional parameters, while OMP requires an expected number of components in the sparse domain; SIRA requires a value for probability *P*; while GDBRA requires four parameters, *p*, *c*, norm selection, and a number of components in the sparse domain (*p* is constant that has values between 0 and 1, while *c* can be 1 for a max, 2 for a min, 3 for a mean, or 4 for a median in generalized deviation calculation). TV minimization requires a number of components in the low and middle frequencies, respectively.

The part for the choice of optimal sigma parameter in HT according to Relation (5) is provided. The sigma can be changed using the scroll button. Moreover, the optimal value, providing the best possible concentration for the selected signal, can be automatically calculated by pressing the *Optimal* button. For other transforms, the value providing the best sparsity is chosen with the help of concentration measure values—the *ℓ*_1_-norm and the Gini index. The reconstruction is initiated by pressing the *Start button*.

Section 2—Graphical presentation of results. This is part of the instrument where the results are graphically presented. Depending on the chosen algorithm, there are several graphics within this section, as presented in Figure 2 (block denoted by number 2). For the ECG signal, there is a possibility to test the reconstruction on the whole signal, but also to choose and observe the QRS complexes using the scroll button. Here, the original and recovered signal are displayed. Moreover, red marks on the selected QRS complex denote the available samples. The transformation can be calculated and displayed for the available samples only, as shown in the panel. The reconstructed sparse representation is presented in the special graph within this section.

**Figure 3:**
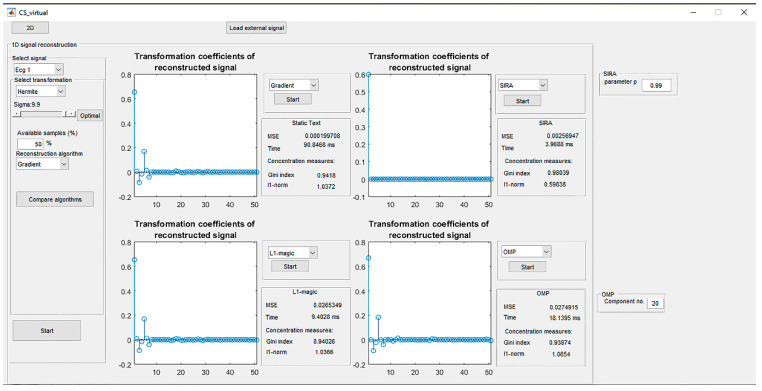
The outlook of the Comparison of the algorithms part applied to the QRS signal recovery.

Section 3—Statistical analyzer: An important part of the proposed instrument is the statistical analyzer part. Here, the concentration measures by the *ℓ*_1_-norm and Gini are calculated for the available samples of the signal, as well as for the signal after the reconstruction. After the signal is recovered using the chosen algorithm, the MSE between the original and reconstructed signal (calculated in the time domain) is displayed on the last part of the statistical analyzer. Concurrently, the execution time of the algorithm is also presented in the same panel. Figure 3 shows the performance of the statistical analyzer demonstrated on the example of the gradient-based reconstruction algorithm.

Example in Figure 2 shows the whole ECG signal on the upper left graph, while the selected QRS complex is shown in the upper right graph. The scroll button below the QRS graph enables QRS selection. HT is selected as a sparsity domain, while the percentage of the available samples is set to 50%. The optimal value for the sigma parameter is used, calculated according to (5). The graph of selected QRS shows also the available samples denoted by red marks. Two lower graphs show HT transform of the available samples (left lower graph) and (sparse) HT of the reconstructed signal. The statistical analyzer displays concentration measures, *ℓ*_1_-norm, and Gini index: (1) measures are calculated using available samples of the selected QRS and (2) using the reconstructed signal. It can be seen that *ℓ*_1_-norm has a lower value for reconstructed signal compared with the value when it is calculated for available samples only, while the value of the Gini index is higher for the recovered signal, as expected.

Section 4—Compare algorithms: The virtual instrument is designed in a way to provide a comparison of the reconstruction results achieved by different algorithms. This is an important part of the instrument, as it enables testing the most suitable approach for a considered biomedical signal or image, in terms of reconstruction quality and processing speed. For this reason, an additional set up section is included and it provides the selection of parameters for the comparative algorithm. This part of the instrument, denoted by 4, is used to select one of the offered algorithms as well as to set its parameters, as described in Section 1. The reconstruction results are displayed graphically and evaluated numerically through MSE, computational time, and concentration measures. The outlook of this part of the software is shown in Figure 3. In the presented example (Figure 3), for a chosen QRS complex and 50% of available samples, the best reconstruction performance is provided by the Gradient algorithm and *ℓ*_1_-magic algorithms in terms of concentration measures. However, the gradient/based algorithm provides slightly better MSE.

### 4.2. Part 2—Reconstruction of 2D Signals

The second part of the Virtual instrument is designed for image reconstruction. Switching between two parts of the instrument can be done using the ***1D/2D*** button at the top left corner of the instrument. The reconstruction of images is performed by several algorithms. Radial-Fourier uses TV minimization with the 2D DFT as a transform basis. The measurements are collected along radial lines from the 2D DFT domain, while the number of radial lines is set as an input parameter of the algorithm.

TV minimization is used with the 2D DCT transform within the second algorithm from the drop-down menu, where there is a possibility to set the number of low and middle frequency coefficients. Another approach is the 2D gradient algorithm, as this algorithm produces efficient results with natural images even in a very low number of available samples. A variation on the method of multipliers is implemented through the Douglas–Rachford algorithm for biomedical image recovery (the UNLocBoX software is used for Douglas–Rachford algorithm implementation [51]).

**Figure 4:**
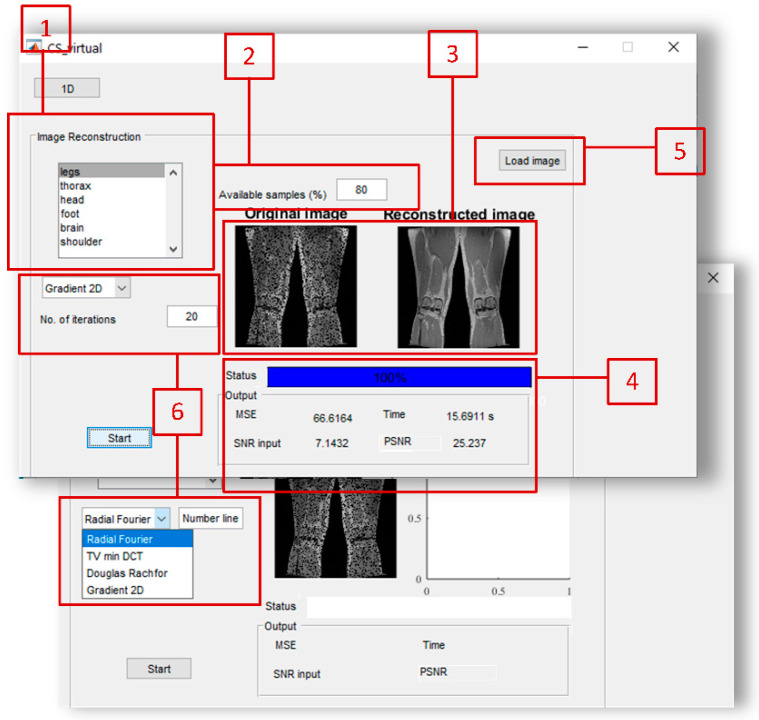
The outlook of 2D part of the proposed Virtual instrument.

The outlook of this part of the instrument is shown in Figure 4. Users can use some of the predefined images, or load their own images. Predefined images can be selected in Section 1. The image database is downloaded from [52].

Section 2: For the selected image, the percent of available samples is selected (i.e., a number of radial lines when considering the radial-Fourier algorithm or number of middle and/or low-frequency coefficients in the TV min approach). The gradient algorithm requires a number of iterations. Section 3: In this section, the original image with missing pixels is shown (left), and the image after reconstruction is performed is shown on the right.

Section 4: Numerical results of reconstruction: Here, MSE, computational time, and input signal to noise ratio SNR and output peak signal to noise ratio PSNR are calculated and displayed. The wait bar is implemented in order to visually show the reconstruction progress. It is important to note that the user can select the arbitrary image that needs to be located within MATLAB m-file (Section 5). Section 6 shows the selection of the algorithms.

## 5. Additional Experimental Evaluation

In this section, some additional reconstruction scenarios are provided and discussed.

**Figure 5:**
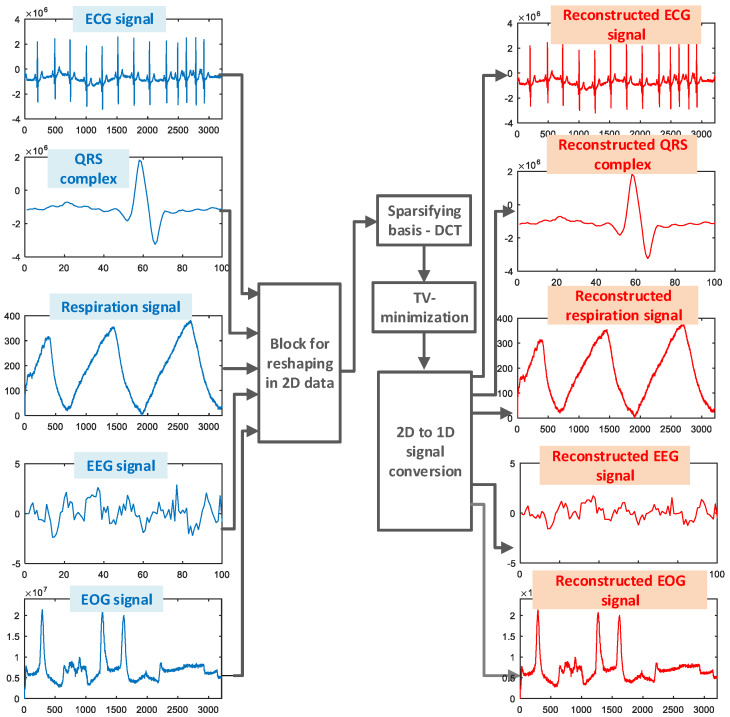
The reconstruction results for different 1D biomedical signals. The total variation (TV) minimization is used and 45% of samples are considered as unavailable. EEG, electroencephalography; EOG, electrooculography.

Example 1: Firstly, the 1D signals are tested. The plots from the virtual instrument are extracted and presented within the diagram in Figure 5. Therefore, the approach that provided successful reconstruction in all considered signals, TV minimization, is used for obtaining the results in Figure 5. Signals for heart rate monitoring, the ECG and the extracted QRS complex; the respiration signal; and the signals for brain activity monitoring, the EEG and EOG signals, are tested. The signal is firstly reshaped from vector to matrix (2D) form. Reshaping is done column-wise. The DCT is considered as a domain of sparsity and the measurements are collected randomly from this domain. The next step is the reconstruction of 2D data using TV minimization, as shown in Figure 5. Finally, the reconstructed signal is back to its 1D form.

**Figure 6:**
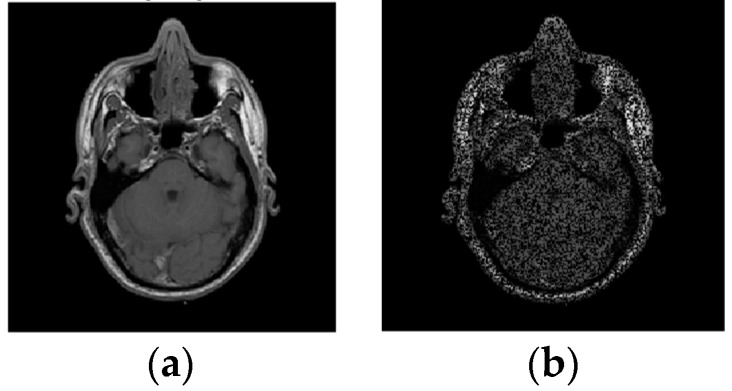
The original (**a**) and image with missing pixels (**b**); 45% of pixels are unavailable.

**Figure 7:**
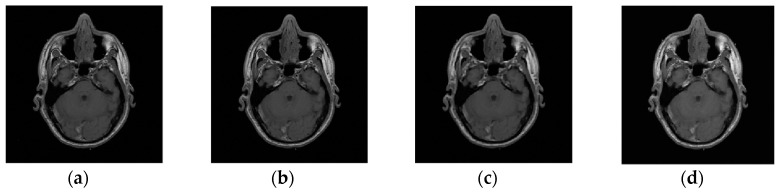
Reconstruction results for the magnetic resonance imaging (MRI) image, considering 45% the missing information; the results are obtained using the (**a**) gradient; (**b**) radial-Fourier; (**c**) TV-min-DCT; and (**d**) Douglas–Rachford algorithms.

Example 2: In this example, the reconstruction efficiency is tested for the MRI image. Unlike the 1D case, the MRI image can be successfully recovered with almost all implemented algorithms. The percentage of missing samples is considered to be around 45. The original and image with missing samples are shown in Figure 6. The reconstruction procedures show that the radial-Fourier provided the best PSNR. However, the processing time is the longest using the radial-Fourier approach. The reconstruction results are shown in Table 1 and Figure 7.

## 6. Conclusions

Virtual instrument for compressive sensing signal reconstruction is proposed. The software is composed for a specific purpose, that is, biomedical signal recovery, considering both 1D and 2D biomedical signals. Several commonly used algorithms for sparse signal recovery are implemented. Additionally, the proposed instrument enables a comparison of different algorithms, where specific parameters can be changed independently for each algorithm. The second part of the instrument is used for image reconstruction. All functions within the instrument can be used, upgraded, or changed with some other algorithms in order to build other application-related instruments for solving specific problems. This software can be a useful tool for clinicians and healthcare professionals in an era when low-power portable medical devices become widely used and when safe and fast communication is of great interest. The part enabling the comparison of algorithms and choosing the most suitable one can be useful in medical practice, as it enables selection of the most accurate and fastest approach.

## Figures and Tables

**Table 1:** The PSNR and reconstruction time for different algorithms. TV, total variation; DCT, discrete cosine transform.

Algorithm	Percentage of Missing Pixels	Reconstruction Time (sec)	PSNR [dB]
Gradient	45%	11.4	30.5
Radial-Fourier	43%	120.1	47.8
TV-min-DCT	45%	0.9	43.9
Douglas–Rachford	45%	11.2	31.5

## References

[1] Donoho D.L. (2006). Compressed sensing. IEEE Trans. Inf. Theory.

[2] Eldar Y.C. (2015). Sampling Theory: Beyond Bandlimited Systems.

[3] Stankovic S., Orovic I., Stankovic L. (2014). An automated signal reconstruction method based on analysis of Compressive Sensed Signals in Noisy Environment. Signal Process..

[4] Stanković S., Orović I., Sejdić E. (2015). Multimedia Signals and Systems: Basic and Advance Algorithms for Signal Processing.

[5] Vulaj Z., Brajović M., Draganić A., Orović I. Detection of Irregular QRS Complexes using Hermite Transform and Support Vector Machine. Proceedings of the 59th International Symposium ELMAR-2017.

[6] Brajović M., Orović I., Daković M., Stanković S. (2018). Compressive Sensing of Sparse Signals in the Hermite Transform Basis. IEEE Trans. Aerosp. Electron. Syst..

[7] Stanković L.J., Brajović M. (2018). Analysis of the Reconstruction of Sparse Signals in the DCT Domain Applied to Audio Signals. IEEE/ACM Trans. AudioSpeechLang. Process..

[8] Foucart S., Rauhut H. (2013). A Mathematical Introduction to Compressive Sensing.

[9] Lakičević M., Draganić A., Orović I., Stanković S. Sparse signal reconstruction using gradient-threshold based method. Proceedings of the 7th Mediterranean Conference on Embedded Computing, MECO.

[10] Rauhut H., Schnass K., Vandergheynst P. (2008). Compressed sensing and redundant dictionaries. IEEE Trans. Inf. Theory.

[11] Draganić A., Orović I., Stanković S. (2017). On some common compressive sensing recovery algorithms and applications—Review paper. Facta Univ. Ser. Electron. Energetics.

[12] Chhatlani A., Dadlani A., Gidwani M., Keswani M., Kanade P. Portable Medical Records Using Internet of Things for Medical Devices. Proceedings of the 8th International Conference on Computational Intelligence and Communication Networks (CICN).

[13] Vuorela T., Seppä V., Vanhala J., Hyttinen J. (2010). Design and Implementation of a Portable Long-Term Physiological Signal Recorder. IEEE Trans. Inf. Technol. Biomed..

[14] Krejcar O., Janckulik D., Slanina Z. The Real Code Migration on portable devices for personal biotelemetric systems. Proceedings of the PORTABLE-POLYTRONIC 2008—2nd IEEE International Interdisciplinary Conference on Portable Information Devices and the 2008 7th IEEE Conference on Polymers and Adhesives in Microelectronics and Photonics.

[15] Paradiso R., Loriga G., Taccini N. (2005). A wearable health care system based on knitted integrated sensors. IEEE Trans. Inf. Technol. Biomed..

[16] Sejdic E., Can A., Chaparro L.F., Steele C.M., Chau T. (2012). Compressive sampling of swallowing accelerometry signals using time-frequency dictionaries based on modulated discrete prolate spheroidal sequences. Eurasip J. Adv. Signal Process..

[17] Donoho D.L. (1995). De-noising by soft-thresholding. IEEE Trans. Inf. Theory.

[18] Candes E.J., Romberg J., Tao T. (2006). Robust uncertainty principles: Exact signal reconstruction from highly incomplete frequency information. IEEE Trans. Inf. Theory.

[19] Draganić A., Orović I., Stanković S., Zhang X., Wang X. Compressive Sensing Approach in the Table Grape Cold Chain Logistics. Proceedings of the 6th Mediterranean Conference on Embedded Computing MECO.

[20] Stankovic L.J., Stankovic S., Orovic I., Amin M. (2013). Robust Time-Frequency Analysis based on the L-estimation and Compressive Sensing. IEEE Signal Process. Lett..

[21] Tropp J., Gilbert A.C. (2007). Signal recovery from random measurements via orthogonal matching pursuit. IEEE Trans. Inf. Theory.

[22] Candes E., Romberg J. L1-Magic: Recovery of Sparse Signals Via Convex Programming. https://statweb.stanford.edu/~candes/software/l1magic/.

[23] Stankovic S., Stankovic L.J., Orovic I. (2014). Relationship between the robust statistics theory and sparse compressive sensed signals reconstruction. IET Signal Process..

[24] Figueiredo M.A., Nowak R.D., Wright S.J. (2007). Gradient projection for sparse reconstruction: Application to compressed sensing and other inverse problems. IEEE J. Sel. Top. Signal Process..

[25] Draganić A., Orović I., Lekić N., Daković M., Stanković S. Architecture for single iteration reconstruction algorithm. Proceedings of the 4th Mediterranean Conference on Embedded Computing MECO.

[26] Stankovic L.J., Stankovic S., Amin M. (2014). Missing samples analysis in signals for applications to L-estimation and compressive sensing. Signal Process..

[27] Zhao D., Zhao F., Gan Y. (2020). Reference-Driven Compressed Sensing MR Image Reconstruction Using Deep Convolutional Neural Networks without Pre-Training. Sensors.

[28] Draganić A., Orović I., Stanković S., Li X., Wang Z. (2017). An approach to classification and under-sampling of the interfering wireless signals. Microprocess. Microsyst..

[29] Rao Y., Zhao G., Wang W., Zhang J., Jiang Z., Wang R. (2019). Adaptive Data Acquisition with Energy Efficiency and Critical-Sensing Guarantee for Wireless Sensor Networks. Sensors.

[30] Yin Z., Lu X., Chen W. (2018). Echo Preprocessing to Enhance SNR for 2D CS-Based ISAR Imaging Method. Sensors.

[31] Kimmel R., Tai X.C. (2019). Processing, Analyzing and Learning of Images, Shapes, and Forms: Part 2. Handbook of Numerical Analysis Series.

[32] Stanković L.J., Daković M., Vujović S. (2014). Adaptive variable step algorithm for missing samples recovery in sparse signals. IET Signal Process..

[33] Dong W., Wu X., Shi G. (2014). Sparsity Fine Tuning in Wavelet Domain with Application to Compressive Image Reconstruction. IEEE Trans. Image Process..

[34] Wu J., Liu F., Jiao L.C., Wang X., Hou B. (2011). Multivariate Compressive Sensing for Image Reconstruction in the Wavelet Domain: Using Scale Mixture Models. IEEE Trans. Image Process..

[35] Najarian K., Splinter R. (2012). Biomedical Signal and Image Processing.

[36] Lustig M., Donoho D., Pauly J.M. (2007). Sparse MRI: The Application of Compressed Sensing for Rapid MR Imaging. Magn. Reson. Med..

[37] Tao Y., Rilling G., Davies M., Marshal I. (2013). Carotid blood flow measurement accelerated by compressed sensing: Validation in healthy volunteers. Magn. Reson. Imaging.

[38] Brajovic M., Orovic I., Dakovic M., Stankovic S. (2017). On the Parameterization of Hermite Transform with Application to the Compression of QRS Complexes. Signal Process..

[39] Sandryhaila A., Saba S., Püschel M., Kovačević J. (2012). Efficient Compression of QRS Complexes Using Hermite Expansion. IEEE Trans. Signal Process..

[40] Sucic V., Saulig N., Boashash B. (2011). Estimating the number of components of a multicomponent nonstationary signal using the short-term time-frequency Rényi entropy. EURASIP J. Adv. Signal Process..

[41] Hurley N., Rickard S. (2009). Comparing Measures of Sparsity. IEEE Trans. Inf. Theory.

[42] Zonoobi D., Kassim A.A., Venkatesh Y.V. (2011). Gini index as sparsity measure for signal reconstruction from compressive samples. IEEE J. Sel. Top. Signal Process..

[43] Zonoobi D., Kassim A.A. (2014). On ECG reconstruction using weighted-compressive sensing. Healthc. Technol. Lett..

[44] Tageldin M., Al-Mashaikki T., Bali H., Mesbah M., Cheng L., Leung A., Ozawa S. (2018). EEG Sparse Representation Based Alertness States Identification Using Gini Index. Neural Information Processing. ICONIP 2018. Lecture Notes in Computer Science.

[45] Chen C.-L., Chuang C.-T. (2017). A QRS Detection and R Point Recognition Method for Wearable Single-Lead ECG Devices. Sensors.

[46] Vulaj Z., Draganić A., Brajović M., Orović I. A tool for ECG signal analysis using standard and optimized Hermite transform. Proceedings of the 6th Mediterranean Conference on Embedded Computing, MECO.

[47] Stanković I., Brajović M., Daković M., Ioana C., Stanković L.J. (2020). Quantization in Compressive Sensing: A Signal Processing Approach. IEEE Access.

[48] Eckstein J. (1992). On the Douglas-Rachford splitting method and the proximal point algorithm for maximal monotone operators. Math. Program..

[49] MIT-BIH ECG Compression Test Database. http://www.physionet.org/physiobank/database/cdb.

[50] The BioSig Project. /http://biosig.sf.net/.

[51] UNLocBoX, Matlab convex optimization toolbox. https://epfl-lts2.github.io/unlocbox-html/.

[52] National Library of Medicine. https://www.nlm.nih.gov/research/visible/mri.html.

